# Bioelectric Fields at the Beginnings of Life

**DOI:** 10.1089/bioe.2022.0012

**Published:** 2022-12-15

**Authors:** Alistair V.W. Nunn, Geoffrey W. Guy, Jimmy D. Bell

**Affiliations:** ^1^ Research Centre for Optimal Health, Department of Life Sciences, University of Westminster, London, United Kingdom.; ^2^ The Guy Foundation, Chedington, Dorset, United Kingdom.

**Keywords:** bioelectricity, origins of life, thermal vents, mitochondria, quantum mechanics, thermodynamics

## Abstract

The consensus on the origins of life is that it involved organization of prebiotic chemicals according to the underlying principles of thermodynamics to dissipate energy derived from photochemical and/or geochemical sources. Leading theories tend to be chemistry-centric, revolving around either metabolism or information-containing polymers first. However, experimental data also suggest that bioelectricity and quantum effects play an important role in biology, which might suggest that a further factor is required to explain how life began. Intriguingly, in the early part of 20th century, the concept of the “morphogenetic field” was proposed by Gurwitsch to explain how the shape of an organism was determined, while a role for quantum mechanics in biology was suggested by Bohr and Schrödinger, among others. This raises the question as to the potential of these phenomena, especially bioelectric fields, to have been involved in the origin of life. It points to the possibility that as bioelectricity is universally prevalent in biological systems today, it represents a more complex echo of an electromagnetic skeleton which helped shape life into being. It could be argued that as a flow of ions creates an electric field, this could have been pivotal in the formation of an energy dissipating structure, for instance, in deep sea thermal vents. Moreover, a field theory might also hint at the potential involvement of nontrivial quantum effects in life. Not only might this perspective help indicate the origins of morphogenetic fields, but also perhaps suggest where life may have started, and whether metabolism or information came first. It might also help to provide an insight into aging, cancer, consciousness, and, perhaps, how we might identify life beyond our planet. In short, when thinking about life, not only do we have to consider the accepted chemistry, but also the fields that must also shape it. In effect, to fully understand life, as well as the yin of accepted particle-based chemistry, there is a yang of field-based interaction and an ethereal skeleton.

## Introduction

In this article, we suggest that a missing factor in origins of life theories is that a flow of ions, for instance in a deep-sea thermal vent, generated an electric field, which led to a far from equilibrium dissipative self-organizing structure and a prototypical morphogenetic field putting bioelectricity center stage in the origin and evolution of life. This might suggest that the smallest quanta of life, at least on this planet, is a self-replicating and adaptive structure capable of maintaining a self-reinforcing biofield that enables the dissipation of an energy gradient, which, critically, holds information about its overall shape. From this perspective, the uncoupling of ion gradients and futile cycling can perhaps be viewed as mechanisms not only to enable dissipation, but also to maintain these fields, and so fulfil the arrow of entropy.

In relationship to genetics, it would suggest that the blueprint to build field generating structures was a latter evolutionary strategy born out of a field-driven condensation of information holding molecules that enabled true life to replicate and move beyond its birthplace. This was probably driven by changes in the local environment, in effect stress that forced natural selection.

In the “A Quantum Thermodynamic Perspective of Life” section, we review the concept that life can be explained from a quantum thermodynamic perspective, and then in the “Morphogenetic Fields and Life: An Old Idea” section, review how this leads onto why electric fields could well be pivotal. In the “Life's Origins and Charged Particle Flow” section, we then use these ideas to provide a perspective on how fields could have been pivotal in life's origins due to the movement of ions, which, potentially, favor metabolism first and thus alkaline thermal vents, and in the “From Thermal Vents to Ion Channels; In An Early Biological Dissipative Fröhlich Condensate?” section, we review how both quantum mechanics and thermodynamics could lead to more complex protein structures, which could be viewed as forms of a “Fröhlich condensate” as self-organizing resonant dissipating structures.

In the “From Prokaryotes to Eukaryotes; Cooperation, Ion Channels, and Cytoskeletons” section, we then build on these ideas by reviewing evolution from prokaryotes to eukaryotes, in particular, how the inter-relationship between fields and protein structures was key in the development of cooperativity and complexity. Finally, in the “The Ethereal Skeleton at the Beginning of Life: Conclusions and Implications” section, we discuss how these concepts could be integrated into a morphogenetic theory of life, and what the implications might be for aging, life definition, astrobiology, uncoupling, death, viruses, and the origins of cancer.

## A Quantum Thermodynamic Perspective of Life

“What is life?” is a question that has been posed by many, including one of the founding members of quantum mechanics, Schrödinger.^1^ In fact, discussions on the role of “significant” quantum effects in biology were also undertaken by Niels Bohr and Pascual Jordan in the 1930s.^2,3^ This is perhaps hardly surprising, as it is generally agreed that the best description of our universe, and thus the life within it, is based on quantum mechanics and quantum field theory (QFT).^4^ However, before quantum mechanics, the discipline of thermodynamics was developed ostensibly to help better understand steam engines, but soon led onto concepts like entropy.^5^

Ever since quantum mechanics was developed after Einstein suggested, in answer to the black body radiation problem that electromagnetic (EM) radiation was quantized, the two ideas existed side by side and were often treated as separate subjects; more recently, however, with the development of new technologies, the field of quantum thermodynamics is now striving to bring the two together.^6^ In fact, the concept of dissipative adaption of thermodynamic systems is now being extended into the quantum realm, hinting at a quantum thermodynamics of driven self-organization.^7^

Overall, it is now becoming broadly accepted that thermodynamics must have played a role in the origins of life,^8,9^ as has a role for quantum mechanics and a universal mechanism of charge transport.^10^ In that light, perhaps one of the most famous quotes in biology, attributed to Albert Szent-Györgyi that “life is nothing but an electron looking for a place to rest,” which captures the importance of charge flow perfectly and has been used in origins of life theories,^11^ was certainly prescient as the movement of charge creates a field and is fundamental to quantum mechanics.

## Morphogenetic Fields and Life: An Old Idea

Thus, as life can be defined as a structure that dissipates energy by channeling ions down gradients, the fields this generates could also be part of a homeostatic feedback system as they, in turn, influence the movement of the charge. In fact, the idea that electric fields are important in biology is far from new.

At around the same time as quantum mechanics was being developed in the early 20th century, others, such as Alexander Gurwitsch, were trying to answer fundamental biological questions about how the shape and growth of organisms were controlled. This gave rise to the idea of morphogenetic fields and the role of bioelectromagnetic and photobiological factors in the structural organization of biosystems. This has since grown to embrace many different scientific disciplines, including quantum mechanics, nonequilibrium thermodynamics, order out of chaos theories, and self-organizational dissipative theories as proposed by Ilya Prigogine. It also gave rise to the ideas of Herbert Fröhlich about the role of biological coherence and condensates, as well as the potential importance of photonic resonance. In short, the “morphology” of life is perhaps shaped by fields, rather than genetics (this subject is reviewed in more depth in “Fields of the Cell,” edited by Fels et al.^12^).

## Life's Origins and Charged Particle Flow

As the “A Quantum Thermodynamic Perspective of Life and Morphogenetic Fields and Life: An Old Idea” sections indicate, as biology is all about charge flow, we cannot ignore the role of electric fields due to basic quantum thermodynamic principles. If so, when did charge first start to flow? Did life start, then charge flowed, or did charge flow, which kick started life? This is perhaps one of the most fundamental questions about the origins of life and whether or not electric fields may have been pivotal.

With regard to the more “standard” origins of life theories, there are many, ranging from metabolism, to proteins, to lipids, to nucleic acid first informational ideas, with initial power sources including sunlight and geothermal. Most start with simple chemicals^13^; in effect, life arose from geology. The debate had tended to revolve around whether the chemoautotrophic theory on the origins of life is stronger than the heterotrophic “organic soup” idea due to the nature of the free energy sources that drove the earliest anabolic reactions.^14^ Hence theories on the origins of life tend to broadly break down into phototrophic (driven by solar potential and the dissipation of energy as heat in oceans e.g., Michaelian^15^ or photosynthetically active zinc sulfide precipitated on thermal vents, e.g., Mulkidjanian^16^ and Mulkidjanian and Galperin^17^), and geotrophic, with life as a planetary process (driven by geochemical gradients^18^).

Another way of viewing this is that discussions have also tended to revolve around either metabolism or RNA (information) first; however, the “descent of the electron” seems to favor metabolism first. This is perhaps reinforced by the fact that all the starting ingredients can be made on earth, or in outer space—including many aromatic compounds.^11^ Although the metabolism first might favor thermal vents, others also suggest that a composite theory, such hydrothermal impact crater-lakes, due to the potential concentration of prebiotic chemicals, may have been a more likely site.^19^

However, one of the strongest theories is based on the observation that modern life uses chemiosmotic coupling and a proton gradient, hinting at the importance of alkaline thermal vents. This is very much a metabolism-first based on geological chemistry/genes later approach. Interestingly, some of the oldest known proteins, such as ancestral ATPases and energy converting hydrogenase (ECH) fit well with this, in particular, as these vents can also result in the formation of lipid membranes involving a process called “thermophoresis.”^20,21^ Furthermore, evidence of reflexively autocatalytic networks has also been identified in microbial metabolism, which is also consistent with an autotrophic origin of life in thermal vents, which seems to continue to suggest that autocatalytic chemical networks preceded proteins and RNA.^22^

In that light, it is perhaps of relevance that a recent article has also identified the ATPase as being a key determinate of tissue regeneration in relationship to a morphogenetic field generation that is conserved across kingdoms.^23^ Also, as suggested in alkaline thermal vent theories of the origins of life,^20^ the F-type ATP synthase seems to have arisen very early in evolution and is conserved across all domains of life.^24^

The alkaline thermal vent concept is based on the acid-base energy gradient present in the immediate post-Hadean—key in this process were catalysts based on iron-sulfur centers and the evolution of molecules such as the flavins that enabled electron bifurcation to occur.^25^ Critically, data now suggest that the modern successors to these, such as ferredoxin and flavin adenine dinucleotide, as well as many other proteins are reliant on electron tunneling to function^26,27^; tunneling in mitochondria may thus be key in the way they work.^28,29^ It also appears that proton tunneling is also a central component of enzyme function.^30^ Hence, it is possible that quantum effects were important in the origins of life.^31,32^

The bottom line is that as a potential starting point, alkaline thermal vents are as good a candidate as any, as extant biochemistry, ranging from energy systems involving acetyl CoA, to Kreb's cycle intermediates, Fe(Ni)S proteins to the central role of a proton gradient, could have evolved from these. Certainly, the flow of ions would have been conducive to the generation of an electric field, and because of the basic quantum nature of charged entities such as protons and electrons and their interaction with electric fields, the emerging consensus that biology is reliant on significant quantum effect would certainly support this. The thinking is now that rather than the “warm and wet” milieu of biology preventing significant quantum effects, it actually enhances it—the environment-assisted quantum transport (ENAQT) concept via a kind of resonance.^33^

## From Thermal Vents to Ion Channels; An Early Biological Dissipative Fröhlich Condensate?

If the alkaline thermal vent idea is correct, then charge flow may well have come first, which meant that the flow of ions could have generated significant fields resulting in an ion/field-based self-organizing dissipative system. This in turn could have acted as a “nucleus” that “condensed” the available molecules that in turn, could have further stabilized it into a structure where the energy was transferred by recognizable chemistry. So, what would have these first structures been like?

Today, it is thought that conditions in alkaline thermal vents could have led to the evolution of energy capturing ion channels, like ECH and ATPase,^20,34^ which could have been embedded in membranes that could have also formed under these conditions.^21^ In effect, some of the earliest “biotic” structures could have been something like an ion channel that “condensed” around some inorganic structure that was flowing ions and was reinforced by electric fields.

Further support for this perhaps comes from the thought that ion channels, in general, are evolutionarily very old as they are highly conserved across all kingdoms—certainly the proton pumping ATPase/synthases can be traced back all the way to the last common ancestor of all extant life, and the more modern complex versions probably arose from gene duplication events.^35^ Voltage-gated ion channels are also universal.^36^ In effect, proteins that can both extract energy from a gradient, as well as sense and control the flow of ions, had probably been a very important step in life's evolution.

**FIG. 1.**
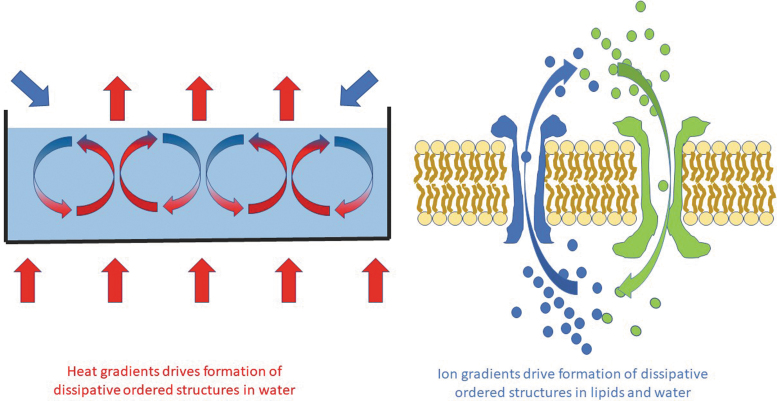
Bénard–Rayleigh cell compared with an ion channel. Although the underlying energy source (might) be different, the appearance of an ordered structure from an apparently chaotic mix of chemicals that enable the dissipation of an energy gradient is perhaps striking. Given that artificial membranes can also spontaneously form pores, then this would add to the concept that what we now define as “life” is actually a more complex version of natural principles definable by thermodynamics—it has, by natural selection of other molecules, built upon a core outcome of order out of chaos that is more robust. It is also possible that this process has relied on manipulating quantum effects.

It could therefore be said that thermodynamics could provide the “drive” for a dissipative structure to form by organizing existing molecules, for instance, in a membrane to form energy extracting and voltage sensing channels. Although potentially on a different scale, the ability of energy to induce a phase transition into an organized dissipative structure such as a Bénard–Rayleigh convection cell,^12^ which looks almost identical to an ion channel, is perhaps striking (Fig. 1). What is also relevant is that membranes can spontaneously form ion channels without the need for any proteins in the presence of an energy gradient; in effect, synthetic lipid bilayers can display many of the effects of say, transient receptor potential channels—especially near to their chain melting temperatures.^37^

But what other physical principles might be at play? As previously discussed, quantum mechanics has of course been long thought to be important in understanding biology.^1,38,39^ In fact, there is an old but highly relevant theory, developed by Herbert Fröhlich, which discusses the role of condensation in biology from the quantum mechanical perspective. In effect, this theory stipulates that energy can be stored in excited vibrational modes in cells by metabolically driven polar oscillating units involving an interaction between the very large EM field generated by a mitochondrion and the cytoskeleton, resulting in a “condensed” energy state in a mode with the lowest frequency.

Critically, the strong electric fields generated by mitochondria could also potentially organize water.^40^ This becomes even more relevant, in that it is entirely possible, as explained by quantum electrodynamic (QED) theory, that water itself can become coherent under certain conditions, for instance, near hydrophilic surfaces under the influence of electric fields and especially when it contains impurities. This is known as exclusion zone (EZ) or interfacial water (reviewed in chapter 5 of Fels et al.^12^). It has even been said that the coherent quantum frequencies of water itself could be pivotal in life.^41^

It is thus perhaps relevant that recent data suggest the presence of an ordered water channel in ATP synthase, which via a Grotthus mechanism, could channel protons.^42,43^ Furthermore, the presence of ordered water in the proton channel of a V-ATPase has also been inferred.^44^ Thus, the possible presence of ordered water that could enhance proton transport in such ancient and important proteins could hint at some kind of resonant condensation process involving electric fields that could be an echo of the very earliest structures as life began.

## From Prokaryotes to Eukaryotes; Cooperation, Ion Channels, and Cytoskeletons

If life is dependent on a charge flow-field interaction, how might this express itself as biology evolved ever greater complexity? A key facet of this maybe that increasing complexity enabled more information to be stored, enhancing the ability to adapt to changing environments. Clearly the evolution of genes was one answer to this, but information can also be stored in networks, enabling rapid responses, for instance, in the interactive flow between ions and the fields they generate.

Data indicate that life was prokaryotic for billions of years until the union of bacteria and Archaeans led to the modern eukaryote.^45^ The evolution of eukaryotic multicellularity, with a means of coupling bioelectric networks, say, via gap junctions, does indicate that this cooperation was certainly a key mechanism for enhancing robustness and storing information.^46^ However, perhaps less appreciated is that cellular cooperation evolved long before this, and seems to have been the normal state of affairs for billions of years in the prokaryotic world: for instance, large colonies of prokaryotes seem to recapitulate phylogeny as they grow.^47^ Critically, ion channels could well be key in this cooperation.^48^ This raises the rather intriguing possibility that not only were fields essential at the beginning of life, but also that this led to cooperation. In essence, life did not evolve as a single cell, but as a cooperating group.

As discussed, ion channels appear to be pivotal in bioelectricity and are very old. However, another emerging evolutionary story is also coming to the fore, and that is prokaryotes also have cytoskeletons made up of precursors to those found in modern cells, such as tubulin and actin—which self-assemble to form polymers.^49^ Critically, it seems that some of these cytoskeletal elements also have enzyme function, suggesting evolution from dual-role proteins—in effect the ability of enzymes to form these polymers.^50^ Today, theoretical research based on the ideas of Fröhlich seems to indicate that these microtubules, certainly in eukaryotes, can exhibit super-radiance and coherent energy transfer, in effect, excitonic resonant states that are modulated by reactive oxygen species (ROS), calcium, and light, and many other chemical parameters; alterations in this system could underlie many pathological states.^51^ Furthermore, static magnetic fields can also affect their polymerization,^52^ hinting at another factor that may have been important.

In terms of more conventional biochemistry, it is well described that tubulin interacts with the voltage-dependent anion channel, a pivotal mitochondrial protein, and modulates its membrane potential—and may well play a key role in cancer.^53^ The key thing here is that mitochondria and the cytoskeleton are intimately and dynamically linked in function and can be spread throughout the cell—right up to the plasma membrane; certainly in neurons, they are pivotal in synapse function, for instance, for energy and calcium homeostasis.^54^ It now seems that not only can gap junctions be pivotal in transfer of mitochondria to other cells,^55^ but also via cell to cell tunneling nanotubes—which appear to be extensions of the cytoskeleton and could well be part of an electrical signaling system.^56^

The underlying premise here is that microtubules, as well as actin and other polymers, especially if coupled to structures which generate very large electrical potentials, such as mitochondria, or any other membrane which can generate both a static, as well as an oscillating electric field and say, voltage sensitive ion channels, could be part a self-organizing field-based dissipating structure. The antecedents of this system would thus come from prokaryotes, which certainly contain all the right ingredients (chemiosmotic coupling, gradients, ion channels, cytoskeleton, etc.)—as well as being highly cooperative. Although morphogenetic bioelectric field research is focused more on ion channels and gap junctions in more modern cells, as cells can be viewed as coupled resonant structures, it would seem logical that any ion channel, or gap junction, is going to be electronically coupled to other structures near it—which must include the cytoskeleton and mitochondria.

This then leads us back to one of the most difficult questions in biology, what is consciousness and how do anesthetics work? Although there are several classical ones, quantum-based theories are being considered. Single-celled eukaryotes display a high level of intelligence and adaptability, which may involve their cytoskeleton as a kind of processing unit; one explanation of anesthesia is that it involves mild disruption of microtubules—and these drugs can inhibit single-celled creatures.

However, these drugs do have many other targets as well. Emerging theoretical calculations are supporting the observation that magnetic fields can influence microtubule structure, and that this is related to a quantum property called spin and a radical pair mechanism.^57^ This would of course fit well with the idea of scale-free cognition as proposed by Levin.^58^ It would also fit with field-based theories of the mind.^59^ Interestingly, some anesthetics also inhibit signaling in plants, such as in the Venus fly trap, which do not have neurons, but do have ion channels^60^—perhaps hinting at a more ancient common mechanism.

It would therefore seem that as complexity arose, the original simpler systems would have formed the basis of larger and larger entities and become more robust. The composite integration of their manifold bioelectric fields not only provides shape, but perhaps also the intelligence that all organisms display—with perhaps the highest expression being consciousness and awareness.

## The Ethereal Skeleton at the Beginning of Life: Conclusions and Implications

**FIG. 2.**
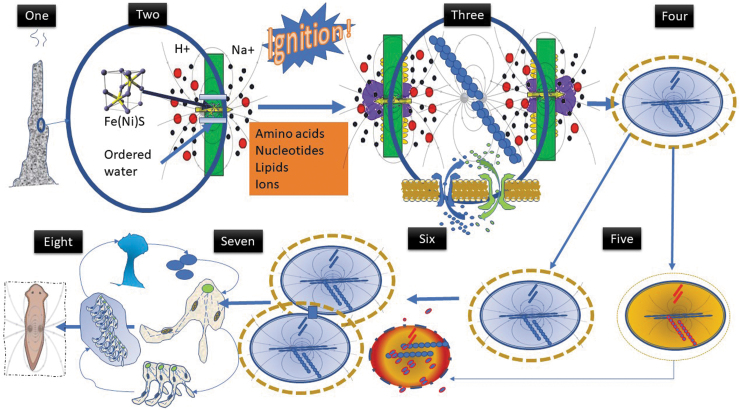
The field quanta ignition of life theory underlying origins of morphogenetic fields. (1) Crystalline cell within thermal vent with ions flowing around and through providing vectored fields, possibly aided by photons generated in the vent resulting in a kind of coherent cavity. (2) Existing chemicals organized into structures that enhance dissipation, engenders chemistry leading to lipids and basic peptides—an “abiotic” self-organizing dissipative structure. (3) Fields enable informational storage and structural polymerization, further “condensing” information—life is “ignited.” (4) Changes in alkaline thermal vent force natural selection and evolution toward independent life utilizing alternate energy sources, becomes autotrophic; emergence of self-contained prokaryotic cell generating own fields enables more complex electrophoretic control of components and maintenance of own proton and ion gradients. In coordination with ion channels, field/cytoskeleton pivotal in cellular intelligence, and environmental sensing. (5) Excess energy forces growth as a means of dissipation, but due to decrease in internal order and natural thermodynamic principles, fission results in new, more stable entities, and natural selection continues to act. Uneven fission ensures removal of less efficient components—aging related to reducing dissipation and field maintenance: death occurs when field collapses. (6) Under greater stress, less efficient “units” die to enable survival of more robust “units,” enhancing cooperation, in which fields and contact points play key role in coordination. (7) Further changes in environment and evolution result in formation of new type of cell, the eukaryote, which capitalizes on the ability to use oxygen and unleash far more free energy and thus complexity, encompassing cooperation, cell death, and differentiation (e.g., dictyostelium). (8) Fields play a key role in controlling shape of emerging multicellular life. The field concept could then begin to explain the emergence of evermore complex informational computational structures, such as the brain.

In summary, the movement of any charged entity, whether it be an electron, proton, or large ion, will create a field as it moves, and then an electrostatic field if there is an uneven charge distribution across a barrier. Conditions characterized by the movement of ions may well have been present in something like an alkaline thermal vent on the immediate post-Hadean earth resulting in large electrochemical potential. These fields could have been pivotal in organizing the chemicals and water present to form far from equilibrium dissipative structures. Critically, these new structures could have held information about the environment and due to natural selection could have started to evolve, leading to increasing complexity, cooperation, adaptability, and robustness (Fig. 2).

If extant life does harbor an ethereal field skeleton as an echo of life's beginnings, what might it tell us? For instance, does it shed light on aging, a definition of life, quantum effects in biology, biological uncoupling of gradients, death, viruses, astrobiology, and even the origins of cancer?

### Aging; death of the field quanta, long live the field quanta

If life started as a kind of negative entropic dissipative “vortex” reinforced by information containing electric fields, it might suggest not only a minimal “quanta” of life, but also a limited existence if its constituents degraded. Critically, because a damaged unit could potentially damage other units (hence stopping dissipation), for instance via oxidative or reductive stress, controlled termination could have been selected for, as, by doing this, it enabled other systems to survive and therefore maintain dissipation. It perhaps also redefines what we mean by “inflammation”; if it cannot be fixed, remove it, but with the novel viewpoint that it is acting at the global level—everything, from the scale of molecules all the way up to whole species is replaceable, as long as life itself survives.^61^

The new field of quantum dissipative adaptation does suggest that this approach is applicable to life.^62^ In this regard, controlled death is actually extremely ancient, and evolved in prokaryotes.^63^ This implies that “dying for the greater good” has probably been around since life started.

One way to view this could be related to Le Chatelier theories on networks under perturbation, where some parts of the network fail, and the system reroutes to maintain stability.^64^ On the largest scale, that of the earth, life can thus be viewed as the fourth geosphere, in effect, every subcomponent, from molecules, all the way up to species is disposable, but is part of adaptive dissipative system called life.^18^ It has been suggested that a key component of the ability of cells to organize and adapt to stress is via forming bioelectric networks.^58^ In this sense, bioelectricity transfers information about individual “quanta” of life and how well they are dissipating energy: when they start to fail, selection removes them. Aging and death of individual components are enshrined in the process, of which the loss of the ability to maintain a bioelectric field must be central.

### A bucket full of chemicals is not life…

The ethereal skeleton concept underlying the origins of the morphogenetic field suggests not only what we might define as life, but perhaps also the order in which things occurred at the beginning. Clearly a bucket full of chemicals is not life, even if they are the right ones. They need organizing and be able to continually dissipate energy and have the capacity to renew when broken. Even if you put electric currents through this bucket of chemicals and get some reorganization and chemistry, you do not get life. The chemicals need to be organized and this seems to have been a sticking point for many theories on the origins of life. However, if we think of information in fields, then we may have a way out of this.

In terms of electrodynamics, the shape is information as dictated by the flow of charged particles and thus vectored fields. The field could thus exert “force” on any charged molecules, and thus provide a mechanism for condensing them into a shape, which could provide a self-reinforcing structure to dissipate energy by enhancing a flow of ions—but the most stable would be those that enhance properties that say, catalyze reactions that further enhance dissipation—such as charge separation, in effect, a stable microstate.

Now of course, this could just exist as a quasi-stable state if nothing changed, but if some memory of the structure could be created, for instance, via a polymer that was organized by the field that enabled the molecule to persist during natural variations that would normally result in dissolution of the structure, this could provide something for natural selection to work on—especially if these field enhanced critical quantum effects like water order. The likelihood of this also increases with the advent of self-organizing membranes driven by entropy.

It is beyond the scope of this article to go into greater detail about the fine detail of the origins of life, but the concept that the shape of a field was determined by ion flow does suggest that “information” could have been a starting point that helped organize metabolism—but it may not have required specific “information molecules” to evolve first. It would suggest that they coevolved through natural selection to provide the building blocks to maintain the field memory of the shape. This shape is still seen today, but in a vastly more complex form, for instance, in the V-ATPase, and of course up the scale to individual cells, and multicellular organisms such as ourselves.

However, it is possible that “true cellular life” began when multiple energy extracting voltage sensitive ion channels combined to provide a more intricate field that “remembered” the most efficient dissipative alignment. This might suggest that enzymatic polymers may have been a very early part of life, which eventually became a cytoskeleton, which, of course, is key in cellular shape. Although it is probably almost impossible to ascertain what polymers may have existed at the beginning, the ability of many molecules to form polymers is perhaps suggestive. Key in this is that they may have started out with one function, but with time, they adapted to another role. In short, many of the polymers we see today, from DNA to tubulin, may have had quite a different role billions of years ago.

### Field ontogeny recapitulates field phylogeny: proof of significant quantum effects in biology?

In a way, it could be argued that the “ethereal skeleton to the morphogenetic field” paradigm might also be called “field ontogeny recapitulates field phylogeny.” This of course does strongly suggest the extent that biology might be using significant quantum effects, especially if approached from the QFT concept.

What is becoming clear is that the electron transport chain and its components seem to be reliant on quantum effects such as tunneling. For example, it is the role of FeS proteins with specific quantum spin properties interacting with the emerging importance of ordered water that could be key.^65^ Of particular relevance is the application of QED theory and the ability of EM fields to interact near charged surfaces to induce coherence resulting in EZ (ordered) water that could have profound and organizing effects on chemistry in biology, in particular, for instance, aiding tunneling (chapter 5 in Fels et al.^12^). Indeed, the principle of ENAQT is becoming increasingly recognized as playing a role in biology, for instance, in exciton transfer in chromophore chains.^33^

Some authors are claiming that, at least in modern cells, it is likely that the interaction between the electron motive force generated by mitochondria, microtubule oscillations, and the induction of water order is essential in cell health, and when mitochondria malfunction, it can lead to conditions like cancer.^66^ Certainly, mitochondrial function has long been thought to play a key role in aging, with maintenance of the ETC being pivotal; this is borne out by recent parabiosis data whereby the introduction of young blood into an aged organism prominently upregulates components of the ETC in multiple organs, leading to a healthier phenotype.^67^

As previously discussed, a key link between fields and function is also suggested by the role of quantum spin; for example, the role of photons, cryptochromes, and ROS production and navigation, as well as perhaps a far more basic role in controlling oxidative stress via spin-correlated radical pairs influenced by magnetic fields.^68^ Although it could be argued that evolution may have selective enhanced and amplified quantum effects to improve biological function once life started, it may also be possible that it was a key requisite right at the beginning.

### An additional role of uncoupling; generation of electric “memory” fields

There is perhaps another aspect to life that could be linked to bioelectricity, and its creation, and that is uncoupling. Uncoupling, in biology, is generally regarded as the process whereby the generation of high energy chemicals, such as ATP, is uncoupled from an ion gradient produced by the flow of electrons down an electron transfer chain; the most common ion is of course a proton, but many other ions are also “recycled.” The process has been described as “wasteful,” as 30% or more of the energy is apparently lost, even in prokaryotes, reducing growth by as much as three times. Although it clearly has roles in signaling, heat generation and modulating oxidative stress,^69,70^ it could also be argued that it represents a dissipative process that generates electric fields.

This of course brings us back to a good candidate for a structure that could have generated consistent electric fields on a prebiotic earth: an alkaline thermal vent fed by a constant flow of ions. Semiconducting compounds, such as FeS, would have been key in enabling flow of electrons, while the fields generated could have influenced potentially important quantum effects, such as water order that would have aided proton movement. The charge separation so generated would also act to concentrate prebiotic compounds so enabling a dissipative, and perhaps, coherent “condensate” to form that according to thermodynamics, would become self-organizing, especially if some kind of resonating cavity was formed.

Indeed, the ability of some bacteria to generate excitons in very low levels of light has been used to investigate quantum effects in biology by exposing them to quantized light in an optical cavity light system; the data suggest that they could become entangled, and amazingly, they remain alive.^71^ Critically, thermal vents emit light, particularly at the longer wavelengths.^72^

So, although highly speculative, the generation of light in thermal vents could have also played a role in generating coherent structure. The fields so generated could react, and hold, information about the environment, so giving rise to the very first morphogenetic fields—they would also be able to act as electrophoretic guides. In this model, organization of “memory” molecules would follow as they would allow information to be captured and retained and, despite the energy cost, would enable natural selection and increased dissipative efficiency that would drive complexity according to the information cycle of Brillouin.^73^

Although it could be predicted that one of the very first structures to evolve would be an ion channel, which would be in keeping with several other theories on the origins of life, molecules that were key in shape, such as the precursors to microtubules, could also have been pivotal. This very much points toward “fields before genetics.”

### Elsewhere: thoughts on astrobiology

The “ethereal skeleton” concept may help in the search for life beyond our planet. As has often been said, looking for it depends on what we think we are looking for, which itself depends on our definition of life and thus where we should look. As is probably entirely predictable because we tend to base our ideas on our own biology, is it carbon based, and does it require water? What about the energy source or temperature range, and would it require an electron acceptor? The concept of a field-based life form might give us some further clues. It might provide us with an insight that different sets of molecules, but still organized by a field, could well be life if they could replicate and evolve.

### Death and viruses: defining life

We have, in another article, discussed how thermodynamics and the quantum world could, perhaps, explain inflammation and death as inflammation describes a process to try and restore a functional dissipative structure following stress.^61^ When viewed from the field concept, it would suggest that there comes a point when an organism can no longer maintain a dissipative field, either because its structure become badly damaged, or it can no longer repair it, with the latter ability decreasing with age; it therefore dies. However, many organisms, or their eggs/spores, can survive quite harsh conditions. This raises the question of whether biological entities that cannot generate their own fields such as viruses are actually “alive”; do they only become “alive” when in a cell generating a field, or are they never truly alive? Mature human red blood cells do not contain DNA, but most would say they are alive, even if they cannot replicate.

What does seem to be key is that replication of life requires a structure that can, when ready, dissipate energy, but to grow and repair it also requires a polymer containing the instructions to build new bits, which perhaps suggests a key component of the definition life is that it can rebuild damaged components, enabling it to maintain dissipation over extended periods of time and robustness to adapt to variations in the environment.

### A final word: cancer, any clues from the beginnings of life?

It has been suggested that cancer cells exhibit disturbed EM coherence that is related to mitochondrial dysfunction.^74^ It is also known that a key feature of many cancer cells is that they stop communicating properly, which is often associated with changes in gap junction function and connexins; mortality from cancer rapidly increases following metastasis.^75^ It is thus relevant that prokaryotes have precursors to gap junctions,^76^ cytoskeletal components,^49^ and nanotubes.^77^ In short, as discussed in an earlier section, cooperation has been around for billions of years and is perhaps the “norm” rather than a single-celled existence. However, many prokaryotic species do have single-celled, noncooperative stages, which are usually associated with reproduction and survival of harsh conditions. Intriguingly, most eukaryotes also have single-celled stages.

So could evolution be viewed differently, cooperation first, and then survival of species via single-celled dispersal? It could be argued that the polycellular structure of a thermal vent could have been an ideal hive-styled incubator, where life got going, not in an individual cell, but all at once across 1000s of resonating cavities; it is likely that bioelectric communication would have been essential for this. Although these alkaline thermal vents can last for 1,000s of years,^34^ plate tectonics and subduction would mean that they would eventually change, applying a selective pressure. Maybe those cells that had evolved to maintain their own field shapes by using alternative energy sources were able to move, either directly, or by going into a state of suspension, and relocate. The resemblance to metastasis is thus uncanny.

The demonstration of the importance of bioelectric fields has led to the concept that these fields might be reprogrammed to control cancer—especially as these fields could define shape and the position of cells in a multicellular organism. Key in this are ion channels.^78^ Further insight may perhaps be gleaned from data that suggest an immortalized cell can undergo oncogenic transformation by upregulating glucose-6-phosphate dehydrogenase activity as it bolsters antioxidant and nucleotide synthesis, tellingly, this can also be mimicked by simply supplying the cell with exogenous antioxidants and nucleosides.^79^ This would suggest that metabolic reprogramming, and bioelectric fields, are closely linked, as the energy state, redox, and ion channel function are all coupled. It might even hint that the ability to make polymers that could act as blueprints for structures was a pivotal event in enabling single cells to survive.

Perhaps the final piece of the puzzle here is that calorie restriction has long been known to have anticancer effects.^80^ In effect, under energy restriction, it appears that cells become more cooperative, but with extra energy, they tend to go off and do their own thing and replicate; thermodynamically, this is just another way of dissipating. Tellingly, many tumors exhibit aerobic glycolysis, the so called “Warburg effect,” which is associated with acidification of their microenvironment, but the cell has a more alkaline interior; this seems to have many benefits for the tumor, including abstraction of energy via ATPases at the plasma membrane.^81^

However, cancer cells still rely on oxidative phosphorylation, and recent data suggest that one of the ways that they can evade the immune system is by inducing immune cells to transfer whole mitochondria to them via nanotubes, effectively hijacking them.^82^ This of course would completely change the bioelectric field around both cells, but it also highlights the well-known importance of mitochondria in cancer metastasis.^83^

Although these ideas are speculative, we may need to think differently about the origins of cancer and the role of electric fields and potentially, mitochondria. In effect, cancer can be viewed as a natural consequence of excessive energy requiring dissipation, which has become coupled to a shift in the bioelectric field that enables a cell to disengage itself from its fellows and become less cooperative, but then perhaps becomes more able to move around. Once it has found a new niche following metastasis, it reengages with the cells in its new environment. Tellingly, this process is well documented to be associated with the suppression of the local immune system; from a bioelectric point view, this is simply reestablishing communications with its neighbors.

Given the large electric fields generated by mitochondria, this might explain their close association with cancer, and perhaps hint that mitochondrial function is coupled to wound resolution and limb regeneration. As has been said, the field density across the inner membrane of a mitochondrion is not far off that found in a bolt of lightning.^84^ At the cellular level, fields are thus very dense.

So what is cancer in these terms? It could represent an outcome from the very earliest days of life where excess energy drove the formation of new dissipative structures that became self-sustaining, as they could maintain form by generation of their own bioelectric templates. However, ultimately, cooperation continued to evolve as it resulted in structures that could dissipate for longer and were more robust due to integration of their bioelectric fields and information. In terms of thermodynamics, life has thus always had to balance dissipation by creation of new compounds and thus replication and growth, versus maintenance of uncoupling to maintain self-sustaining structures. This would suggest that excess calories will increase the incidence of cancer, which is exactly what we see^85^; conversely, burning calories through physical activity is protective.^86^ Movement is of course just another way of dissipating energy, perhaps suggesting why it evolved.

One interpretation of this is that the excess energy stimulates not only growth to dissipate the energy but also a propensity for single cell survival that is coupled to natural selection of cells that become less cooperative. This then becomes “hard wired” through mutation in key pathways that is coupled to alterations in bioelectric fields. This might begin to explain why many secondary plant metabolites have anticancer functions, as they can induce selective uncoupling and dissipation, which evolved from their ability to act as sunscreens.^87^

Finally, an interesting hypothesis falls out of this, and this is that the evolution of eukaryotes was driven by synergizing with the ancestors of the mitochondria to improve the ability to move. This of course strongly suggests that for most eukaryotes, their morphogenetic fields are tightly coupled to mitochondrial function. This might provide us with new avenues in how to tackle cancer.
